# Resveratrol Protects DAergic PC12 Cells from High Glucose-Induced Oxidative Stress and Apoptosis: Effect on p53 and GRP75 Localization

**DOI:** 10.1007/s12640-013-9439-7

**Published:** 2013-11-12

**Authors:** Justine Renaud, Julie Bournival, Ximena Zottig, Maria-Grazia Martinoli

**Affiliations:** 1 Cellular Neurobiology, Department of Medical Biology, Université du Québec à Trois-Rivières, Trois-Rivières, QC G9A 5H7 Canada; 2 Neuroscience Research Unit, Centre de recherche, Université Laval, Ste-Foy, QC G1V 4G2 Canada

**Keywords:** Resveratrol, High glucose, Apoptosis, Oxidative stress, Neuroprotection, GRP75

## Abstract

Resveratrol (RESV), a polyphenolic natural compound, has long been acknowledged to have cardioprotective and antiinflammatory actions. Evidence suggests that RESV has antioxidant properties that reduce the formation of reactive oxygen species leading to oxidative stress and apoptotic death of dopaminergic (DAergic) neurons in Parkinson’s disease (PD). Recent literature has recognized hyperglycemia as a cause of oxidative stress reported to be harmful for the nervous system. In this context, our study aimed (a) to evaluate the effect of RESV against high glucose (HG)-induced oxidative stress in DAergic neurons, (b) to study the antiapoptotic properties of RESV in HG condition, and c) to analyze RESV’s ability to modulate p53 and GRP75, a p53 inactivator found to be under expressed in postmortem PD brains. Our results suggest that RESV protects DAergic neurons against HG-induced oxidative stress by diminishing cellular levels of superoxide anion. Moreover, RESV significantly reduces HG-induced apoptosis in DAergic cells by modulating DNA fragmentation and the expression of several genes implicated in the apoptotic cascade, such as Bax, Bcl-2, cleaved caspase-3, and cleaved PARP-1. RESV also prevents the pro-apoptotic increase of p53 in the nucleus induced by HG. Such data strengthens the correlation between hyperglycemia and neurodegeneration, while providing new insight on the high occurrence of PD in patients with diabetes. This study enlightens potent neuroprotective roles for RESV that should be considered as a nutritional recommendation for preventive and/or complementary therapies in controlling neurodegenerative complications in diabetes.

## Introduction

Glucose is the essential energy substrate of the central nervous system and large amounts of it are required to fill the high energetic needs of neurons. Unlike muscle cells or adipocytes that depend on insulin, glucose uptake in neurons depends mainly on its extracellular concentration (Tomlinson and Gardiner 2008). Persistent episodes of long-term glucose exposure may induce oxidative stress that results in cellular damage (Giaccari et al. 2009), such as neuropathic complications resulting from hyperglycemia in uncontrolled diabetes (Rajabally 2011). Accumulating evidence has enlightened the relationship between diabetes and neurodegenerative disorders, including Alzheimer’s disease (AD) (Vignini et al. 2013) and Parkinson’s disease (PD) (Jagota et al. 2012). Recent literature has reported an increased risk of developing PD in patients with type 2 diabetes mellitus (Hu et al. 2007; Sun et al. 2012).

PD is a neurodegenerative disorder characterized by the progressive loss of nigrostriatal dopaminergic (DAergic) neurons in the *substantia nigra pars compacta* (SNpc). DAergic neurons in this region are selectively lost due to the high activity of monoamine oxidase and elevated levels of iron which both lead to increased generation of reactive oxygen species (ROS) (Pearce et al. 1997; Cui et al. 2012). At the cellular level, mechanisms of high glucose (HG)-induced toxicity are similarly sustained by oxidative stress in vitro (Bournival et al. 2012; Cao et al. 2012) as well as in vivo (Styskal et al. 2012). By increasing aerobic respiration, raised sugar metabolism promotes excessive formation of ROS which, jointly with insufficient antioxidant defences, may damage cells (Apel and Hirt 2004). Indeed, generation of mitochondrial superoxide is increased and is thought to be at the origin of several HG-induced complications (Brownlee 2001). Currently, it is well known that oxidative stress may lead to apoptosis (Circu and Aw 2010) and increased production of ROS in HG conditions may account for glucose neurotoxicity duly observed.

In addition, several genes are known to be implicated in the pathogenesis of PD, such as PINK1 and DJ-1. Glucose-regulated protein 75 (GRP75, also called mortalin/mtHSP70/mot-2), a member of the cytoprotective Hsp70 family of chaperons, interacts with both PINK1 (Jin et al. 2006, 2007; Li et al. 2005; Rakovic et al. 2011) and DJ-1 (Jin et al. 2005; Li et al. 2005). GRP75 is mainly localized within the mitochondria matrix of neurons where it accomplishes several functions such as mitochondrial import and oxidative stress management (Yaguchi et al. 2007). Overexpression of GRP75 leads to extended life span in nematodes and human cells. On the other hand, it serves as a major target for oxidation and it was shown to be involved in aging of nerve cells and in particular in the degeneration of DAergic neurons (Burbulla et al. 2010). In mitotic cells, GRP75 localized in the cytoplasm sequestrates and inactivates p53 preventing its nuclear translocation and apoptosis (Kaul et al. 2001, 2005; Wadhwa et al. 2002). Indeed, p53 is a tumor suppressor protein known to play an important role in evoking apoptosis when located in the nucleus by encouraging the transcription of several pro-apoptotic genes such as Bax (B cell lymphoma 2 [Bcl-2]-associated X protein, Macip et al. 2003). p53 activity is stabilized in response to oxidative stress through posttranslational modifications disrupting interactions with negative regulators (Neilson et al. 2008). It is also a recurrent target in PD given the involvement of oxidative stress in p53 activation (Nair 2006) and the evidence of DNA fragmentation and chromatin condensation in DAergic neurons of the SNpc in PD patients (Hartmann and Hirsch 2001; Tatton 2000).

Prevention of neuronal loss in PD has not yet been addressed by existing symptomatic treatments. Neuroprotection by dietary polyphenols may be an interesting avenue in current attempts to overcome oxidative stress induced by hyperglycemia. We have recently shown that quercetin and sesamin, antioxidant polyphenols, exert neuroprotective effects in neurons exposed to HG (Bournival et al. 2012). The stilbene resveratrol (RESV) is another polyphenol, primarily found in red wine, known for its potent cardioprotective, antiinflammatory, and anticarcinogenic actions (Rosa et al. 2012; Aluyen et al. 2012). Our group, as well as others, has highlighted its potential in defending neurons against oxidative assaults induced by a spectrum of treatments, including neurotoxins (Gélinas and Martinoli 2002; Blanchet et al. 2008; Bournival et al. 2009; Peritore et al. 2012; Wu et al. 2012) or cerebral ischemic injury (Morris et al. 2011; Simão et al. 2012). Abundant literature suggests that RESV plays a protective role in several neurodegenerative diseases including PD, AD, and Huntington’s disease (Albani et al. 2010; Hung et al. 2010) as well as against neuroinflammation (Foti Cuzzola et al. 2011).

Although the beneficial properties of RESV in neurodegenerative diseases are extensively depicted in the literature, its role in defending neurons against HG-induced damage has yet to be elucidated. The present study was designed to examine the neuroprotective effects of the polyphenol RESV in differentiated DAergic PC12 cells maintained in HG condition. NGF-differentiated PC12 cells are a reliable model for the investigation of oxidative stress and neuroprotection of DAergic neurons. They express tyrosine hydroxylase (TH), high affinity dopamine transporter, estrogen receptor-α and -β, neurofilaments, and secrete high levels of dopamine (Kadota et al. 1996; Neilson et al. 2012; Gélinas and Martinoli 2002). In this comprehensive investigation, we outline the roles of RESV in preventing neural parameters of cellular oxidative stress and apoptosis induced by HG exposure in a cellular DAergic system. Our results demonstrate that RESV can modulate the expression and localization of GPR75, and thus might mediate mitochondria pathways of cell stress.

## Materials and Methods

### Drugs and Chemicals

All reagents and chemicals were purchased from Sigma (St. Louis, MO) unless noted otherwise. Mouse anti-GRP75 (raised against amino acids 525–679 of GRP75 of human origin), rabbit anti-p53 (raised against full length p53 of human origin, for immunofluorescence), rabbit anti-Bcl-2 (raised against a peptide mapping at the N-terminus of Bcl-2 of human origin), rabbit anti-Bax (raised against a peptide mapping near the N-terminus of Bax of mouse origin), mouse anticleaved PARP-1 (poly [ADP-ribose] polymerase, raised against C-terminal purified thymus PARP-1 of calf origin), goat anti-HDAC1 (histone deacetylase 1, raised against amino acids 450 to C-terminus of human HDAC1), and mouse anti-GAPDH (glyceraldehyde 3-phosphatase dehydrogenase, raised against recombinant GAPDH of human origin) antibodies were purchased from Santa Cruz Biotechnology (Santa Cruz, CA). Mouse anti-p53 (raised against amino acids surrounding Ser20 of human p53, for Western blotting) and rabbit anticleaved caspase-3 (raised against amino-terminal residues surrounding Asp175 in human caspase-3) antibodies were purchased from Cell Signaling (Boston, MA). Rabbit anti-VDAC (voltage-dependant anion channel, raised against amino acids 152–169 of VDAC of human origin), mouse anti-TH (raised against rat TH) primary antibodies, and anti-mouse and -rabbit horseradish peroxidise-conjugated secondary antibodies were purchased from Sigma. Anti-mouse Cy3 (cyanine 3)-conjugated secondary antibody was purchased from Medicorp (Montreal, QC, Canada). Goat anti-rabbit FITC (fluorescein isothiocyanate)-conjugated secondary antibody was purchased from Millipore (Temecula, CA).

### Cell Culture and Treatments

PC12 cells, obtained from American Type Culture Collection (ATCC, Rockville, MD), were maintained in a humidified environment at 37 °C and 5 % CO_2_ atmosphere. Cells were grown in Roswell Park Memorial Institute medium 1640 (RPMI 1640) supplemented with 10 % (v/v) heat-inactivated horse serum, 5 % (v/v) heat-inactivated fetal bovine serum (FBS), and gentamicin (50 μg/mL). PC12 cell neuronal differentiation was evoked by administration of nerve growth factor-7S (NGF, 50 ng/mL) in Dulbecco’s Modified Eagle medium (DMEM) supplemented with 1 % FBS for 7 days, as already described (Bournival et al. 2009, 2012). The DMEM containing 1.0 g/L of d-glucose (Sigma D5523) is further called control (CTRL) medium, whereas HG DMEM containing 4.5 g/L of d-glucose (Sigma D7777) is named HG medium. DAergic PC12 cells were incubated with CTRL or HG medium for 96 h, unless stated otherwise. We previously performed lactate dehydrogenase-based cytotoxicity assays to determine the appropriate time of treatment in order to study the apoptotic process in the remaining live cells (Bournival et al. 2012). For the last 24 h of treatment, DAergic PC12 cells were incubated with or without RESV (0.1 μM). RESV concentration was selected after dose response and kinetic studies (Bureau et al. 2008; Bournival et al. 2009). An osmotic control consisting of CTRL medium supplemented with 3.5 g/L of D-mannitol (MANN) was used to rule out a hypertonic effect of HG medium on PC12 cells. Charcoal-stripped serum was used in all experiments to ensure that media were free from steroids. For each experiment, initial seeding density was 30,000 cells/cm^2^.

### Detection of Mitochondrial Superoxide Radical

DAergic PC12 cells were grown and treated on collagen-coated circular glass coverslips (Fisher Scientific, Ottawa, ON, Canada). Intracellular superoxide anion (^•^O_2_^−^) production was measured with MitoSOX™ Red (Invitrogen, Burlington, ON, Canada), a fluorogenic dye used for the selective detection of superoxide in the mitochondria of live cells. After treating cells with CTRL or HG medium for 3 h with or without RESV, the cells were incubated with MitoSOX™ Red (5 mM) for 10 min at 37 °C. MitoSOX™ Red is rapidly and selectively targeted to the mitochondria. Once in the mitochondria, it is oxidized by superoxide and exhibits red fluorescence. Cells were washed with Hank’s balanced salt solution (HBSS, Invitrogen), and Hoechst 33342 counterstained all nuclei. Cells were fixed in 4 % paraformaldehyde for 6 min at 37 °C. Coverslips were mounted with Molecular Probes’ ProLong^®^ Antifade Kit (Invitrogen). Images were acquired by a Leica SD AF confocal microscope, and analyzed with Leica Application Suite 3.1.3 software (Leica Microsystems, Concord, ON, Canada). To demonstrate MitoSOX™ Red selectivity, a positive control was performed using sodium diethyldithiocarbamate (DDC), a superoxide dismutase (SOD) inhibitor, in CTRL medium.

### Immunofluorescence and Terminal Deoxynucleotidyl Transferase dUTP Nick End Labeling (TUNEL) Assay

Apoptotic cells were also detected by both TUNEL (Roche Diagnostics, Laval, QC, Canada) assay and activated caspase-3 immunofluorescence. DAergic PC12 cells were grown and treated on collagen-coated circular glass coverslips. Cells were then fixed in 4 % paraformaldehyde for 15 min at 37 °C, washed with phosphate buffered saline (PBS), and further incubated in a blocking and permeabilizing solution (1 % bovine serum albumin [BSA], 0.18 % fish skin gelatin, 0.1 % Triton-X, and 0.02 % sodium azide) for 30 min at RT. Fixed cells were incubated with polyclonal anticleaved caspase-3 antibody 1:500 in PBS overnight. The slides were washed and treated with Cy3-conjugated secondary antibody diluted 1:500 in PBS for 4 h and then incubated with the TUNEL enzyme and fluorescent dUTP mixture for 1 h at 37 °C. Nuclei were counterstained 4′,6′-diamidino-2-phenylindole (DAPI). Coverslips were mounted with ProLong^®^ Antifade Kit. Images were acquired by a Leica SD AF confocal microscope. DAergic PC12 cells were considered to be apoptotic when they were positive for cleaved caspase-3, and their nuclei were stained with TUNEL. The number of apoptotic DAergic PC12 cells among 300 randomly chosen neuronal was counted on 10 different optical fields from three slides per group, as already reported (Bournival et al. 2009, 2012), with Leica Application Suite 3.1.3 software. In each experiment 50 μM Z-DEVD-FMK (Bachem, Torrance, CA), a cell-permeable caspase-3 inhibitor, was used on DAergic PC12 cells in HG and HG RESV conditions as internal control for caspase-3 activation (Bournival et al. 2009, 2012).

### Specific Apoptotic DNA Denaturation Analysis

Specific DNA denaturation in apoptotic cells was assessed with a single-stranded DNA (ssDNA) apoptosis ELISA kit (Chemicon International, Temecula, CA). This procedure is based on the selective denaturation of DNA by formamide in apoptotic cells, but not in necrotic cells (Frankfurt and Krishan 2001). After treatment with CTRL or HG medium with or without RESV, denatured DNA was detected with a monoclonal antibody highly specific to ssDNA and a peroxidase-labeled secondary antibody. The reaction was then stopped with a hydrochloric acid solution, and ssDNA fragmentation was quantified by measuring absorbance at 405 nm with a Multiscan Ascent microplate reader (Thermolab System, Franklin, MA). ssDNA was quantified with reference to CTRL conditions. Absorbance of positive (wells coated with ssDNA) and negative controls (wells treated with S1 nuclease) served as quality control for the ELISA.

### Protein Extraction

DAergic PC12 cells were grown and treated in collagen-coated 6-well plates. Total proteins were extracted using a nuclear extraction kit (Active Motif, Carlsbad, CA). Briefly, cells were washed with a mixture of ice-cold PBS and phosphatase inhibitors, and then harvested in centrifuge tubes. Cell lysis was performed using the supplied buffer, and samples were centrifuged to obtain membrane-free supernatants containing total proteins.

Cytoplasmic-nuclear fractionation was achieved using the nuclear extraction kit. Briefly, cells were washed with a mixture of ice-cold PBS and phosphatase inhibitors, and then harvested in centrifuge tubes. Cytoplasmic membranes were ruptured by treatment with a hypotonic buffer and detergent. Samples were centrifuged to pellet the intact nuclei, and soluble material was preserved as the cytoplasmic fraction. Nuclei were then lysed and conserved in the provided lysis buffer.

Mitochondrial-cytoplasmic fractionation was achieved using a mitochondrial extraction kit (Active Motif, Carlsbad, CA). Cells were washed with a mixture of ice-cold PBS and phosphatase inhibitors, and then harvested in centrifuge tubes. Cells were incubated on ice with isotonic cytosol buffer for 15 min. Cell membranes were ruptured with a pestle homogenizer. Intact cells and nuclei were pelleted after two centrifugations and discarded. Supernatants containing cytoplasm and mitochondria were centrifuged twice to obtain a pellet of mitochondria. The resulting supernatant was preserved as the cytoplasmic fraction. Mitochondria were washed with cytosol buffer and lysed with detergent.

### Electrophoresis and Western Blotting Analysis

Protein dosage was performed with a bicinchoninic acid-based sodium dodecyl sulfate (SDS)-compatible Protein Assay Kit (Pierce, Rockfort, IL) for each fraction of every sample. Equal amounts of protein were loaded onto 12 % SDS polyacrylamide gels. After electrophoretic separation, the gels were transferred to polyvinylidene difluoride membranes (0.22 μm pore size, BioRad, Hercules, CA). The blots were blocked for 1 h at room temperature (RT) in Blotto B (1 % nonfat powdered milk, 1 % BSA, 0.05 % Tween 20, 0.5 mg/mL sodium azide, in Tris buffered saline). Dilution of primary anti-GRP75, anti-p53, anti-Bax, anti-Bcl-2, anti-cleaved PARP-1, anti-GAPDH, anti-HDAC1, anti-VDAC, and anti-TH (1:200, 1:200, 1:50, 1:50, 1:1,000, 1:50, 1:50, 1:500, and 1:10,000, respectively) antibodies was prepared in Blotto B. The blots were then incubated with peroxidase-conjugated secondary antibody (1:10,000) in Blotto B for 2 h at RT and finally developed with an enhanced chemiluminescence substrate solution (Haan and Behrmann 2007).

### GRP75-p53 Colocalization

DAergic PC12 cells were grown and treated on collagen-coated circular glass coverslips. Then, they were fixed in 4 % paraformaldehyde for 15 min at 37 °C, washed with PBS, and further incubated in a blocking and permeabilizing solution for 30 min at RT. Fixed cells were incubated with both rabbit anti-p53 antibody 1:100 and mouse anti-GRP75 1:100 in PBS overnight. The slides were washed with and subsequently treated with anti-rabbit Cy3-conjugated and anti-rabbit FITC-conjugated secondary antibodies both diluted 1:500 in PBS for 4 h. Nuclei were counterstained with DAPI. Coverslips were mounted with Molecular Probes’ ProLong^®^ Antifade Kit. Images were acquired by a Leica SD AF confocal microscope. Colocalization was assessed for 100 randomly chosen DAergic PC12 cells on 6 different optical fields from three slides per group with Leica Application Suite 3.1.3 software.

### Statistical Analysis

Significant differences between groups were ascertained by one-way analysis of variance (ANOVA), followed by Tukey’s post hoc analysis with the GraphPad Instat program, version 3.06 for Windows (San Diego, CA; www.graphpad.com). All data, analyzed at the 95 % confidence interval, are expressed as mean ± standard error of the mean (SEM) from at least 3 independent experiments. Asterisks indicate statistical differences between the treatment and CTRL condition (***p < 0.001, **p < 0.01, and *p < 0.05); plus signs show statistical differences between the treatment and HG condition (^+++^ *p* < 0.001, ^++^ *p* < 0.01, and ^+^ *p* < 0.05).

## Results

### RESV Rescues HG Production of Superoxide

**Fig. 1:**
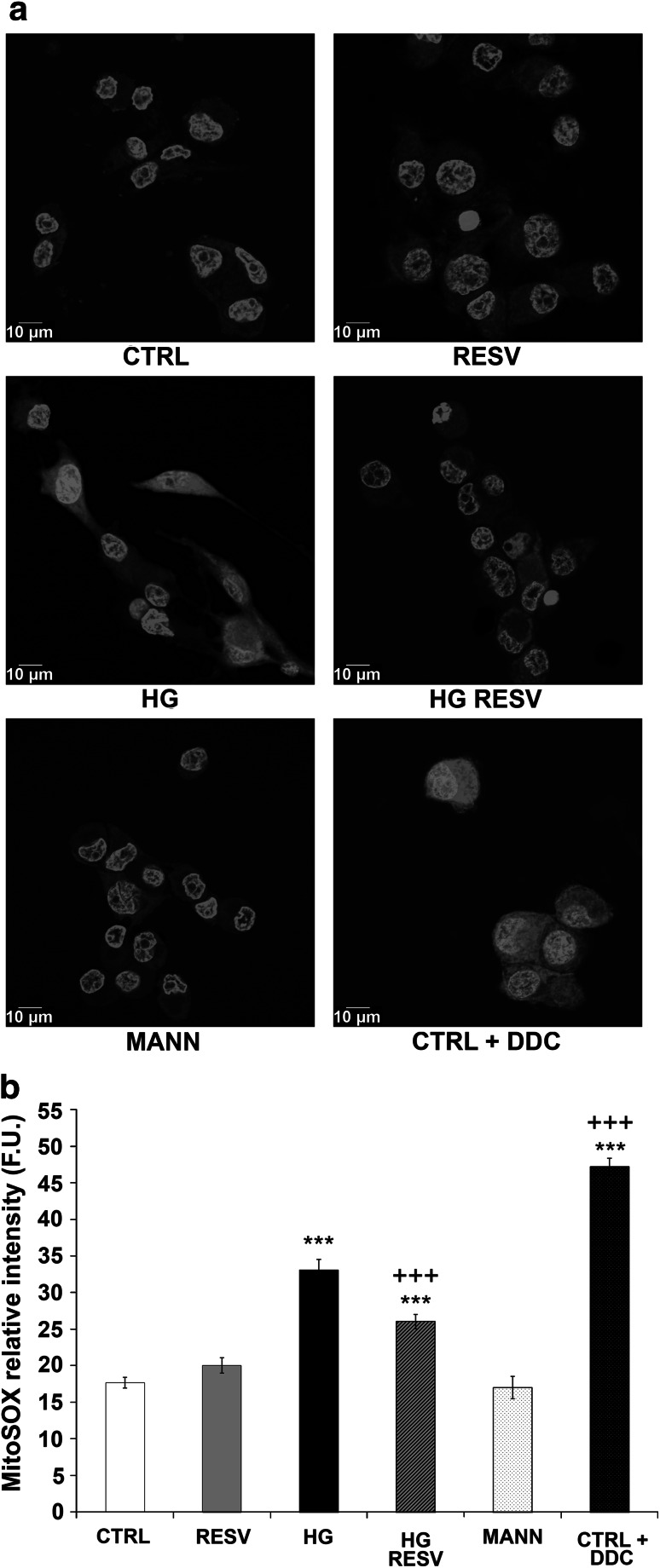
RESV reduces HG-induced superoxide anion production in DAergic PC12 cells. **a** Fluorescence microphotographs. *Blue*: DAergic PC12 nuclei counterstained with Hoechst 33342. *Red*: MitoSOX™ Red superoxide indicator signal. A *marked red signal* is evident in DAergic PC12 cells treated with HG or DDC (CTRL medium + DDC). Red fluorescence was less intense in cells treated with CTRL medium, RESV alone or when RESV was added in HG medium (HG RESV). **b** Semiquantitative image analysis. Fluorescent units (F.U.). ***p < 0.001 compared with CTRL, ^+++^ *p* < 0.001 compared with HG, as determined by one-way ANOVA, followed by Tukey’s multiple-comparison test (Color figure online)

To study the mechanisms underlying the neuroprotective effects of RESV against HG, we measured the production of superoxide with a derivative of ethidium bromide, MitoSOX™ Red, after administration of HG with or without RESV for 3 h. This time period was considered, since free radical generation and eventually oxidative stress are early events in the causative process of cellular death (Zhou et al. 2008; Pérez-De La Cruz et al. 2010, Carange et al. 2011). Figure 1a discloses low fluorescence levels in CTRL and MANN conditions as well as in cells treated with RESV in CTRL medium after 24 h, whereas a marked signal was detected in HG- and DDC-treated cells. When RESV was added to HG medium, fluorescence was strongly reduced. Figure 1b also reports the semiquantitative analysis of mitochondrial superoxide anion presented in Fig. 1a, revealing high fluorescence levels with HG and positive control DDC as well as a very significant reduction (*p* > 0.001) when DAergic PC12 cells in HG medium were treated with RESV. In the DDC condition, inhibition of SOD supports the specific detection of superoxide anion. All nuclei are stained blue by Hoechst 33342 (Fig. 1a).

### RESV Reduces HG-Induced Apoptosis

**Fig. 2:**
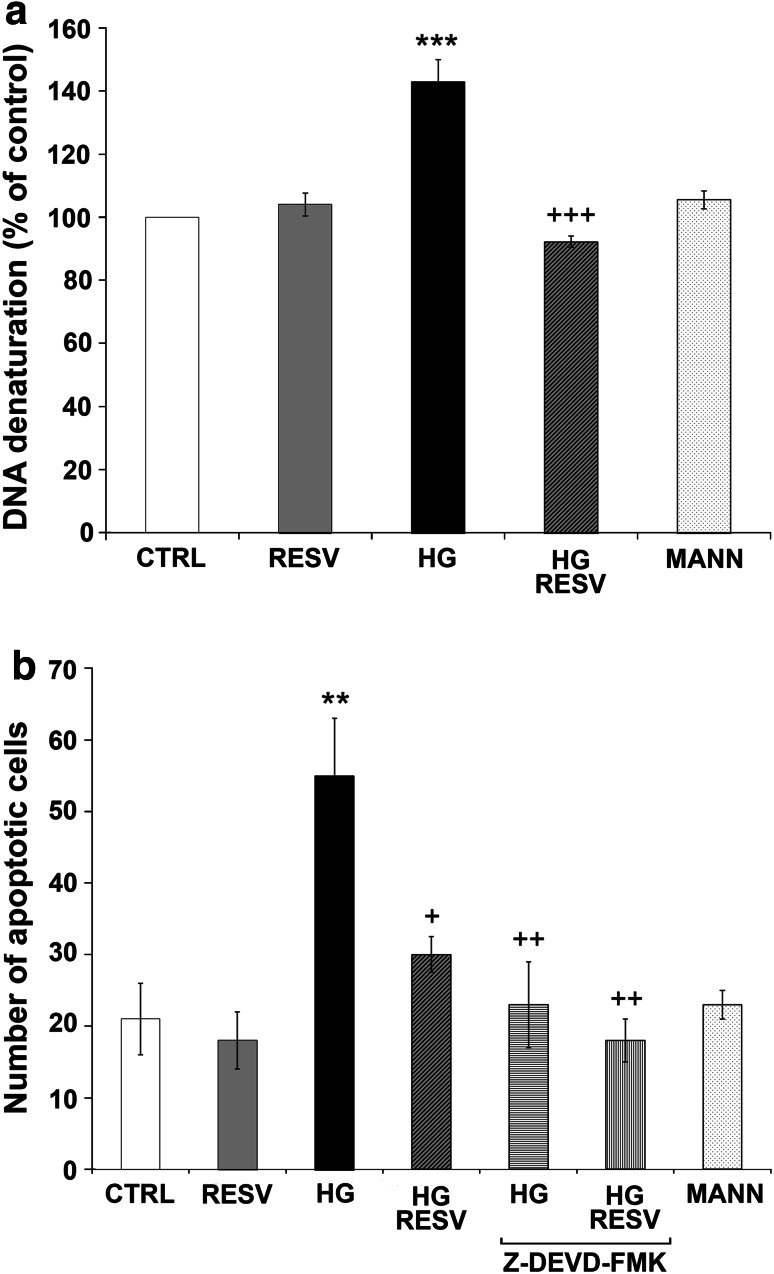

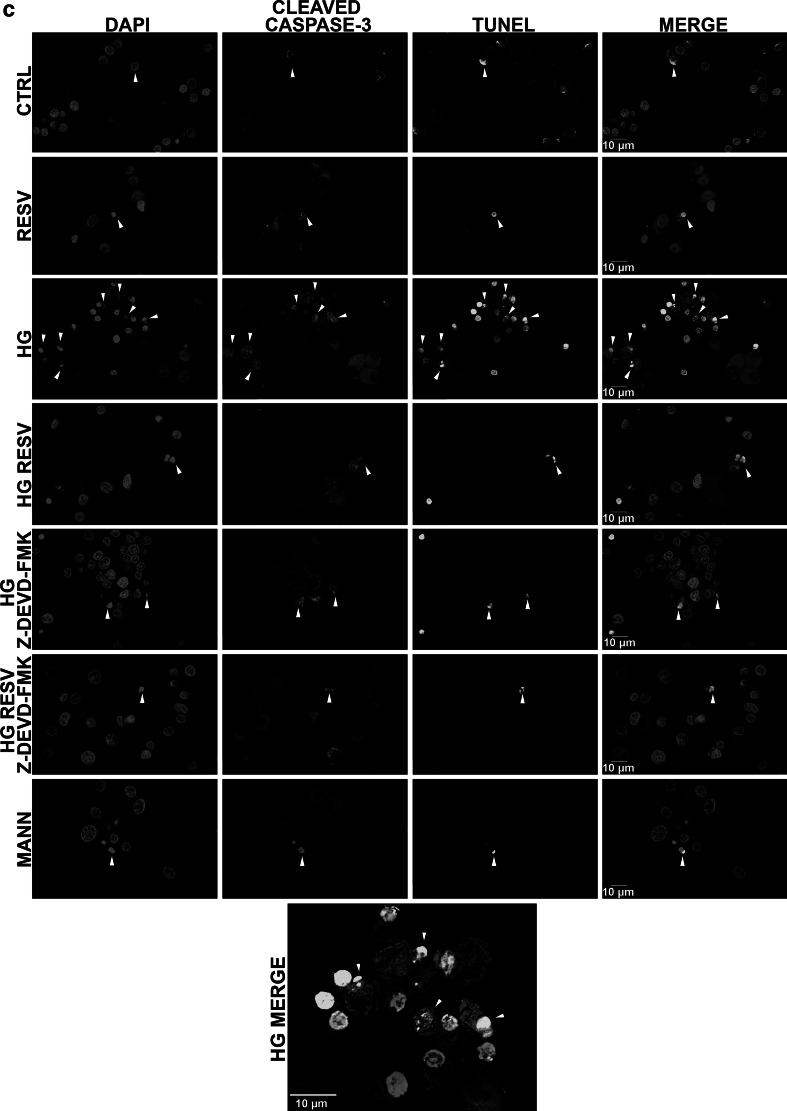
RESV reduces HG-induced apoptosis in DAergic PC12 cells **a** Histogram of specific apoptotic DNA denaturation by formamide in DAergic PC12 cells as detected with a monoclonal antibody against ssDNA. CTRL, MANN, and RESV alone do not affect specific apoptotic DNA denaturation. HG increases apoptotic DNA denaturation. Treatment of HG-exposed cells with RESV elicits a significant decrease in specific apoptotic DNA denaturation (HG RESV). **b** The number of apoptotic DAergic cells among 300 randomly chosen DAergic cells was counted on 10 different optical fields from 3 slides per group, as illustrated in Fig. 2c. **c** Microphotographs of immunofluorescence detection of apoptotic DAergic PC12 cells. *Blue*: DAergic PC12 nuclei counterstained with DAPI. *Red*: anticleaved caspase-3 signal. *Green*: TUNEL staining of nuclei exhibiting DNA fragmentation. Triple-staining (cells points by white arrows) clearly reveals several apoptotic cells on slides treated with HG and fewer apoptotic cells when DAergic PC12 cells are treated with CTRL medium, RESV alone or when RESV is administered in HG conditions (HG RESV). To show that caspase-3 activation is a key step in the HG-induced apoptotic pathway, DAergic PC12 cells were pretreated with 50 μM Z-DEVD-FMK, a cell-permeable caspase-3 inhibitor, followed by treatment with HG, with or without RESV (HG-Z-DEVD-FMK and HG RESV-Z-DEVD-FMK, respectively). MANN condition is similar to CTRL cells. Enlarged microphotograph: HG merge, to show apoptotic nuclei in HG condition. **p < 0.01 and ***p < 0.001 compared with CTRL, ^+^ *p*<0.05, ^++^ *p* < 0.01, and ^+++^ *p* < 0.001 compared with HG, as determined by one-way ANOVA, followed by Tukey’s multiple-comparison test (Color figure online)

We measured DNA denaturation induced by formamide, a specific hallmark of apoptosis (Frankfurt and Krishan 2001), using a ssDNA specific antibody (Fig. 2a). Specific apoptotic DNA denaturation is observed in early as well as in late apoptotic cells. HG condition showed a 43 % increase in apoptotic cells in comparison to CTRL wells. This increment was fully reversed by RESV treatment in HG medium (Fig. 2a). MANN medium did not yield significant apoptosis.

We then examined the effect of HG and RESV on later events of the apoptotic cascade leading to DNA fragmentation. Detection of cleaved caspase-3, the terminal effector caspase responsible for late apoptosis-mediated DNA fragmentation (Fan et al. 2005), was conducted by immunofluorescence alongside a TUNEL assay measuring DNA degradation (Fig. 2b, c). In the HG condition, immunofluorescence revealed the presence of cleaved caspase-3 positive cells (Fig. 2c, red signal), while TUNEL assay stained numerous nuclei undergoing DNA fragmentation (Fig. 2c, green signal). Total nuclei were stained with DAPI (Fig. 2c, blue signal). DAergic PC12 cells were considered to be in late apoptosis when they hosted both caspase-3 activation and DNA fragmentation events (Fig. 2c, cells pointed by white arrows). Treatment with RESV for 24 h clearly reduced the presence of apoptotic nuclei as implied by the lower number of DAergic PC12 cells exhibiting both green and red fluorescence. The number of apoptotic cells was also counted (Fig. 2b), as described in the Materials and Methods section. Administration of RESV decreased the number of apoptotic cells compared to the HG condition. MANN medium did not yield a significant rise in apoptotic cells compared to CTRL. To show that caspase-3 activation is a key step in the HG-induced apoptotic pathway, DAergic PC12 cells were pretreated with 50 μM Z-DEVD-FMK, a cell-permeable selective caspase-3 inhibitor, followed by treatment with HG with or without RESV (Fig. 2b, c).

**Fig. 3:**
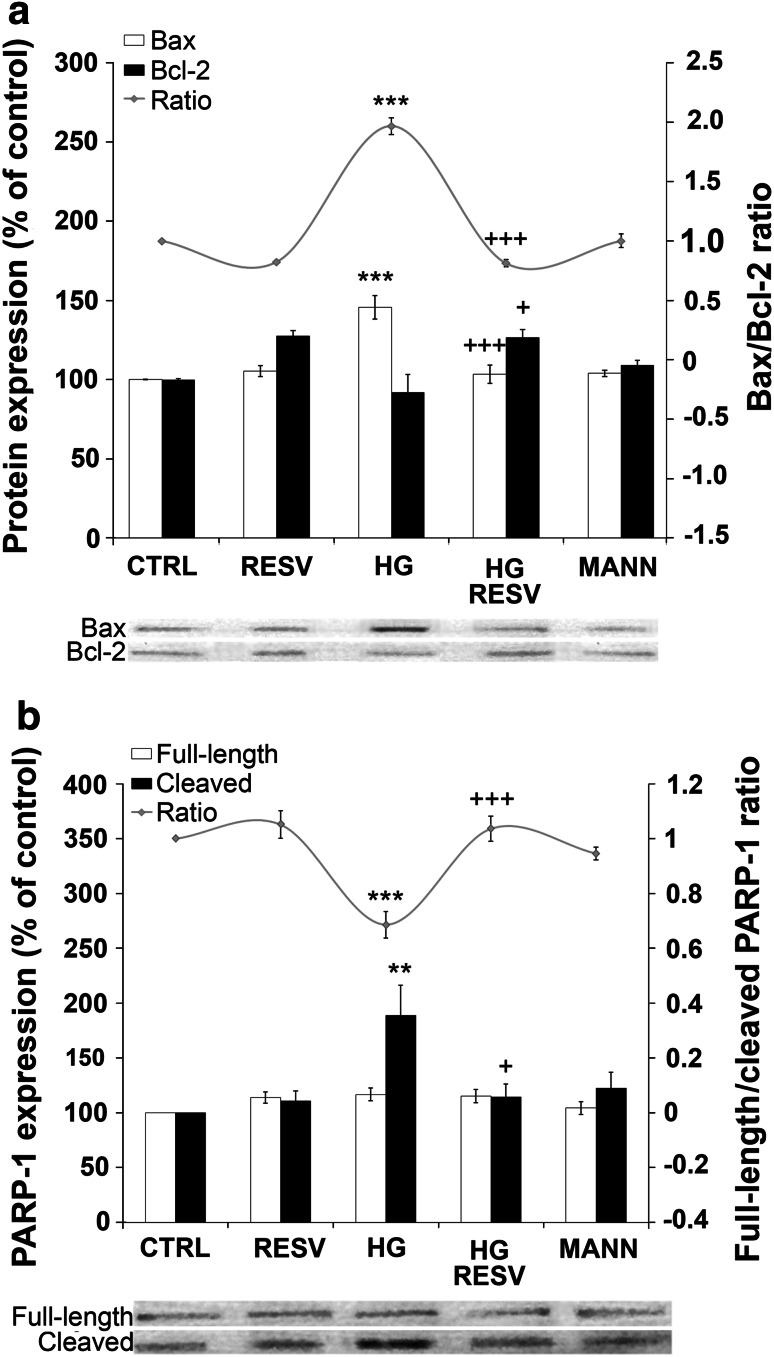
RESV modulates the expression of apoptotic protein markers. **a** Effect of RESV on the Bax/Bcl-2 ratio in DAergic PC12 cells (*full gray line*). CTRL, MANN, and RESV alone do not modulate the Bax/Bcl-2 ratio. HG increases the Bax/Bcl-2 ratio significantly and the addition of RESV to HG medium strongly prevents this increment (HG RESV). *Bottom*: Bax and Bcl-2 bands, as revealed by Western blotting. **b** Analysis of PARP-1 protein expression. These results are presented as the ratio of full-length (*white bars*)/cleaved (*black bars*) PARP-1. CTRL, MANN, and RESV alone do not modulate the PARP-1 ratio in DAergic PC12 cells (*full gray line*). A decrease of PARP-1 ratio is apparent in HG condition. When RESV is delivered in HG condition, a significant increase of full-length/cleaved PARP-1 was evident (HG RESV). Bottom: Western blot bands of full-length and cleaved PARP-1. **p < 0.01, ***p < 0.001 compared with CTRL and ^+^ *p*<0.05, ^+++^ *p* < 0.001 compared with HG, as determined by one-way ANOVA, followed by Tukey’s multiple-comparison test

In order to further support these findings, we analyzed the expression of several proteins acting in the apoptotic cascade. Western blotting was performed on total proteins extracted from DAergic PC12 cells treated with HG or CTRL medium, with or without RESV (Fig. 3). We analyzed the pro-apoptotic Bax and antiapoptotic Bcl-2 protein ratio (Fig. 3a) reported to be correlated with apoptosis (Cory and Adams 2002). A high Bax/Bcl-2 ratio favors the release of mitochondrial factors leading to the activation of effector caspases in the apoptotic cascade (Kang and Reynolds 2009). Our results demonstrate that the administration of HG medium for 96 h increases the Bax/Bcl-2 ratio two-fold compared to CTRL medium, supporting that HG-induced apoptosis in DAergic PC12 cells is mediated, at least in part, by the mitochondrial pathway (Fig. 3a, histogram full gray line). The HG-induced raise of the Bax/Bcl-2 ratio was fully reversed in DAergic PC12 cells treated with RESV. Explicitly, HG medium increases Bax expression (Fig. 3a, histogram white bars, Bax Western bands), but does not modulate Bcl-2 (Fig. 3a, histogram black bars, Bcl-2 Western bands). RESV reverses the HG-induced increase in Bax expression and increases Bcl-2 expression. We also examined the ratio of full-length PARP-1 on inactivated cleaved PARP-1 (Fig. 3b). As Chaitanya et al. (2010) have demonstrated, PARP-1 is a major player in the prevention of programmed cell death and its cleavage by activated caspase-3 is a hallmark of apoptosis. HG treatment markedly reduced full-length/cleaved ratio, which was fully reversed by RESV administered in HG medium (Fig. 3b, histogram full gray line). MANN medium did not have a substantial effect on either the Bax/Bcl-2 ratio or the PARP-1 full-length/cleaved ratio. HG increased PARP-1 cleavage, while RESV prevented this rise (Fig. 3b, histogram black bars, cleaved PARP-1 Western bands). Full-length PARP-1expression was not affected in any condition (Fig. 3b, histogram white bars, full-length PARP-1 Western bands).

### RESV Modulates p53 and GRP75 Subcellular Localization and Colocalization

**Fig. 4:**
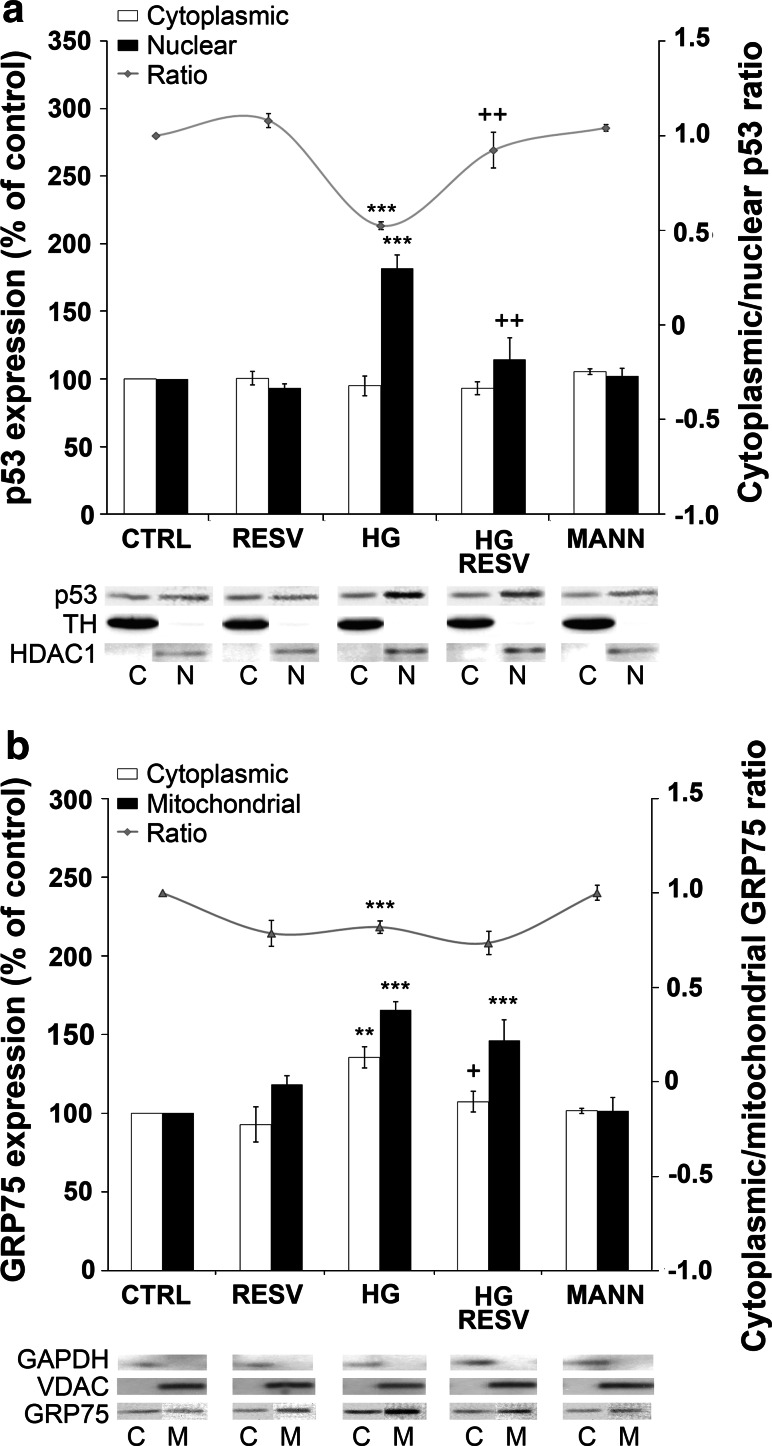
**a** Effect of RESV on the cellular localization of p53 in DAergic PC12 cells. HG medium significantly increases nuclear localization of p53 (*black bars*) and administration of RESV in HG medium prevents this increase (HG RESV). RESV and MANN alone do not modulate p53 cellular localization. Cytoplasmic p53 (*white bars*) is not affected in any condition. Ratio of cytoplasmic/nuclear p53 is decreased in HG condition, which is prevented by RESV administration (HG RESV, *full gray line*). *Bottom*: p53, cytoplasmic fraction purity marker TH and nuclear fraction purity marker HDAC1 bands as revealed by Western blotting. **b** Effect of RESV on the cellular localization of GRP75 in DAergic PC12 cells. HG treatment increases both cytoplasmic and mitochondrial content of GRP75. Administration of RESV in HG medium (HG RESV) significantly reduces cytoplasmic levels of GRP75 (*white bars*), while it does not amend mitochondrial levels (*black bars*). Ratio of cytoplasmic/mitochondria GRP75 is decreased in HG condition, which is prevented by RESV administration (HG RESV, *full gray line*). *Bottom:* GRP75, cytoplasmic fraction purity marker GAPDH and mitochondrial fraction purity marker VDAC bands, as revealed by Western blotting. **p < 0.01, ***p < 0.001 compared with CTRL and ^+^ *p*<0.05, ^++^ *p* < 0.01 compared with HG, as determined by one-way ANOVA, followed by Tukey’s multiple-comparison test

We studied the expression levels of p53, a tumor suppressor, and GRP75, a stress response protein (Fig. 4). In several models, GRP75 binds and inactivates pro-apoptotic p53 in the cytosol, therefore helping to prevent apoptosis. In order to elucidate this alleged relationship between both markers, protein levels were measured in the cytoplasm and the nucleus (p53) or in the cytoplasm and the mitochondria (GRP75) (Fig. 4a and b). Treatment of DAergic PC12 cells with HG medium for 96 h noticeably decreased p53 cytoplasmic/nuclear ratio (Fig. 4a, histogram full gray line). This was prevented by administration with RESV. Expressly, HG increased p53 expression in the nucleus (Fig. 4a, histogram black bars, p53 nuclear Western bands), while it did not seem to affect cytoplasmic levels (Fig. 4a, histogram white bars, p53 cytoplasmic Western bands). RESV in HG medium preserved p53 levels at CTRL range in both compartments. HG administration for 96 h increased GRP75 expression both in the cytoplasm and in the mitochondria (Fig. 4b, histogram white and black bars, GRP75 mitochondrial, and cytoplasmic Western bands). Treatment with RESV in HG medium prevented GRP75 levels from rising in the cytoplasmic fraction only. The result is a small, but significant decrease in the GRP75 cytoplasmic/mitochondrial ratio (Fig. 4b, histogram full gray line). MANN medium did not affect the expression of either GRP75 or p53.

**Fig. 5:**
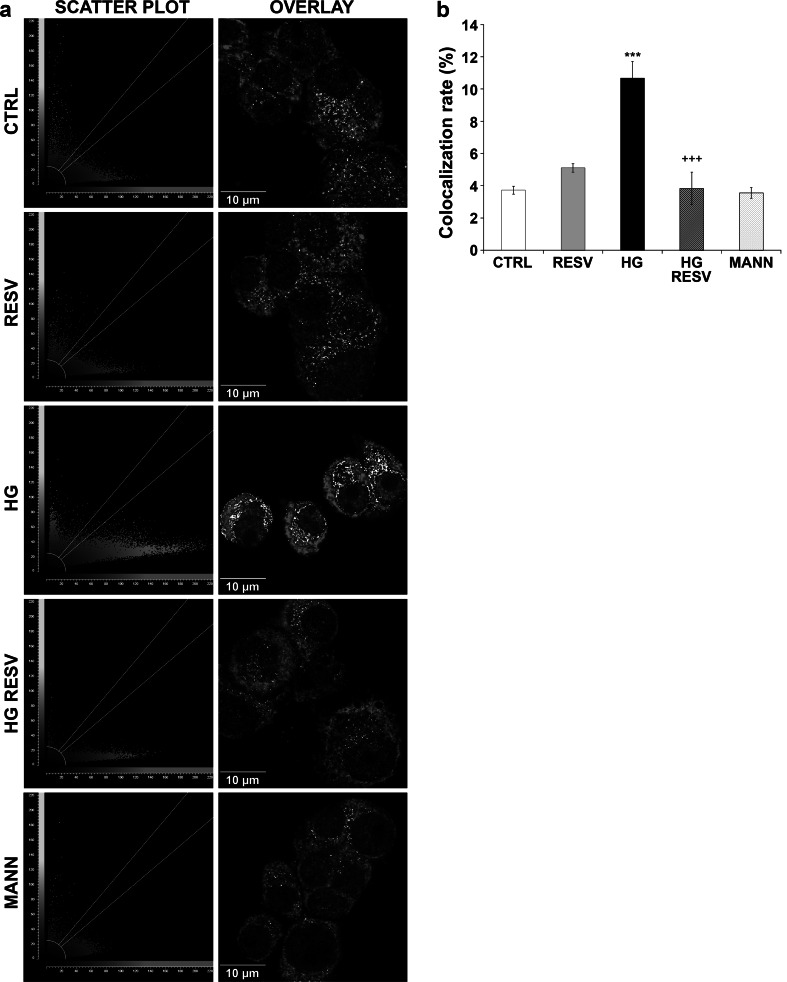
RESV modulates p53 and GRP75 colocalization. **a** Scatter plots and corresponding overlaid micrographs of p53 and GRP75. Scatter plots show signal intensity for p53 (*green signal*) on the *y*-axis and GRP75 (*red signal*) on the *x*-axis. *Each dot* represents one event of fluorescent signal. Thresholds for *green* and *red* signal are optimized at 85 % (*two white lines*) and mean background at 10 % (*arched delimitation*), in each condition. Signal is colocalized when signals in scatter plots are located between the threshold lines on the outside of the background delimitation. *White signal* in microphotographs indicates high probability of colocalization. CTRL, RESV, and MANN conditions are similar in that p53 and GRP75 colocalization is scarce and scattered in the cell cytoplasm. HG increases the white signal around the perinuclear area as well as the number of dots in the region of interest on the plot. Treatment with RESV yields a scatter plot and overlaid white signal (HG RESV) similar to the CTRL condition, suggesting its potential to diminish colocalization between GRP75 and p53. **b** Histogram depicting the colocalization rate of GRP75 and p53 observed in Fig. 5a. HG increases the colocalization rate significantly. RESV administration in HG medium reverses this increase in colocalization rate (HG RESV). All other conditions are similar to CTRL. ***p < 0.001 compared with CTRL and ^++^ *p* < 0.001 compared with HG, as determined by one-way ANOVA, followed by Tukey’s multiple-comparison test (Color figure online)

Finally, to evaluate the potential for GRP75 and p53 to interact in the cytoplasm, immunofluorescence colocalization measurements were performed following treatment of DAergic PC12 cells with HG medium with or without RESV. Scatter plots show that p53 (Fig. 5a, green signal distribution) and GRP75 (Fig. 5a, red signal distribution) signals are mainly independent from one another except for slight colocalization (Fig. 5a, plots and micrographs, Fig. 5b). However, treatment with HG medium still appears to yield more colocalization on the scatter plot (Fig. 5a, plots), which is also supported by the colocalization rate histogram (Fig. 5b). Overlaid pictures of p53 and GRP75 staining (Fig. 5a, micrographs) in CTRL, MANN, and RESV condition show dispersed punctual staining (white signal) in the cytoplasm, while in the HG condition a perinuclear dense staining is clearly visible. Administration of RESV in HG medium reveals a more scattered staining than in HG condition alone.

## Discussion

We previously reported that several natural polyphenols, including the stilbene RESV, exert powerful neuroprotective activity in DAergic PC12 cells against the oxidative burden triggered by the administration of the potent parkinsonian toxin 1-methyl-4-phenyl-1,2,3,6-tetrahydropyridine (MPTP) in vivo (Blanchet et al. 2008) or its active metabolite 1-methyl-4-phenylpyridinium (MPP^+^) in vitro (Gagné et al. 2003; Lahaie-Collins et al. 2008; Bournival et al. 2009). Since hyperglycemia has also been listed as a growing risk factor for PD (Hu et al. 2007; Jagota et al. 2012; Sun et al. 2012), we focused our study on the neuroprotective effect of RESV on HG-induced oxidative stress and apoptosis in DAergic PC12 cells with regard to GRP75 and p53 localization.

The Diabetes Control and Complications Trial (1993) together with the U.K. Prospective Diabetes Study (1998) have determined that hyperglycemia is the culprit to blame for tissue damage in type I and type II diabetes. Currently, we know that overproduction of superoxide is the single upstream event leading to the following pathways involved in glucose toxicity (Giacco and Brownlee 2010): (1) increased flux of glucose and other sugars through the polyol pathway; (2) increased intracellular formation of advanced glycation end products (AGEs); (3) increased expression of the receptor for AGEs and its activating ligands; (4) activation of protein kinase C (PKC) isoforms; and (5) overeactivity of the hexosamine pathway. The formation of AGEs and activation of AGE receptors (Shaikh and Nicholson 2008), the activation of PKC (Aoki and Li 2011), and the dysfunction of the polyol pathway (Ahmed et al. 2009) have been identified as contributors in the development of PD. These mechanisms suggest a strong link between neuronal apoptosis observed in PD and hyperglycemic damage in diabetes (Li et al. 2002, 2008; Klein et al. 2004).

In this study, we demonstrated the defensive role of RESV in counteracting cellular distress parameters evoked by HG in DAergic PC12 cells. We tested NGF-differentiated PC12 cells, a known, reliable, and efficient model for the investigation of oxidative stress, apoptosis, and neuroprotection of DAergic neurons (Gélinas and Martinoli 2002; Lahaie-Collins et al. 2008; Bournival et al. 2012). Since oxidative stress is an essential factor in glucose toxicity and in the pathogenesis of PD, we investigated whether RESV protects DAergic PC12 cells by reducing levels of mitochondrial superoxide in HG condition. Our results show that RESV effectively diminishes superoxide production after as early as 3 h suggesting that oxidative damages occurs upstream of apoptosis. In PD, superoxide reacts with iron cations to form hydroxyl radical (Ramasarma 2012), known to exert very deleterious effects on DNA, lipids, and proteins. This ROS can also react with nitric oxide, an important signaling molecule in the brain, to form peroxynitrite, a powerful oxidant shown to play a significant role in protein aggregation pertinent to PD (Danielson and Andersen 2008).

It is currently well known that oxidative stress may cause apoptosis through several pathways: (1) ROS-induced expression or activation of nuclear factor-kappa B (NF-κB) (Gloire et al. 2006); (2) mitochondria-mediated cell apoptosis (Circu and Aw 2010); (3) ROS-mediated DNA damage and p53 activation (Liu and Xu 2011); and (4) stress-activated protein kinases pathway to apoptosis (Johnson and Nakamura 2007). We performed a set of experiments to investigate the apoptotic cascade in DAergic PC12 cells ensuing oxidative stress to further demonstrate the preventive role of RESV. A specific apoptotic DNA denaturation assay demonstrated that RESV significantly prevents apoptosis in cells exposed to HG. We further examined markers of late apoptosis to determine whether the protein cascade leads to terminal events such as the irreversible fragmentation of DNA. RESV in HG clearly reduced the number of apoptotic PC12 cells in comparison to the HG condition alone as shown by the decline in TUNEL and cleaved caspase-3 double-positive cells. Another target of activated caspase-3 is PARP-1, a protein known to participate in the repair of damaged DNA (Wang et al. 2012). Our findings reveal that the PARP-1 protein ratio, full-length versus cleaved, was decreased after HG treatment and was then improved by RESV administration, hence supporting once more the neuroprotective antiapoptotic role of RESV in a HG paradigm. In addition, the Bax and Bcl-2 expression were studied to determine the apoptotic events surrounding the mitochondria. Bax contributes to the leakiness of the outer mitochondrial membrane, while Bcl-2 blocks the permeability transition pore, thus inhibiting mitochondria-mediated programmed cell death (Smith et al. 2008). The rise in the Bax to Bcl-2 ratio is a characteristic feature in apoptosis (Cory and Adams 2002) equally observed in glucose toxicity (Allen et al. 2005) and in several models of PD including human postmortem brains (Vila and Perier 2008). Our data reveal that the Bax/Bcl-2 protein ratio is increased after HG administration, and is decreased by RESV treatment in the HG condition, strongly suggestive of a role for mitochondrial dysfunction in the mechanisms underlying the apoptosis of DAergic neurons in our cellular paradigm of hyperglycemia.

GRP75 has often been linked to PD pathogenesis as reported in studies showing binding properties to PD-associated proteins in the mitochondria (Li et al. 2005; Jin et al. 2005, 2006, 2007; Rakovic et al. 2011) and reduced levels of the protein in postmortem PD brain samples (Jin et al. 2005; Shi et al. 2008; Burbulla et al. 2010). While GRP75 is mainly confined to the outer membrane of mitochondria, several studies have shown that it may bind and sequestrate pro-apoptotic p53 in the cytosol thereby preventing its entry in the nucleus, impeding apoptosis and ultimately promoting p53 degradation by the MDM2 proteasome degradation pathway (Kaul et al. 2001; Wadhwa et al. 2002; Kaul et al. 2005). Such studies were mainly conducted in cancer cells (Kaul et al. 2001, 2005; Wadhwa et al. 2002) or in naive, mitotic PC12 cells (Guo et al. 2009; Li et al. 2011). Our results obtained in post-mitotic PC12 cells, show that HG treatment increases GRP75 expression in the cytoplasm as well as in mitochondria thus suggesting that GRP75 is induced by HG cellular stress. While RESV reduced GRP75 levels in the cytoplasm, it did not ensure a significant effect in diminishing mitochondria GRP75 localization. Apparently, in our cellular paradigm, RESV modulates the subcellular distribution of GRP75 by preventing cytoplasmic levels from rising. RESV may be responsible for quenching HG-induced stress signals that promote the induction of GRP75 in the cytoplasm. Moreover, p53 localization is increased in the nucleus, which points toward a pro-apoptotic effect of HG on DAergic PC12 cells. RESV in HG medium maintains the cellular distribution of p53, which partially accounts for its antiapoptotic properties. Altogether, these results show an increase of GRP75 in the cytoplasm, while p53 levels rise significantly in the nucleus in HG condition, suggesting relatively weak interaction between both markers in post-mitotic cells. Colocalization studies deepened our understanding of the relationship between GRP75 and p53 in our cellular model. We show that GRP75 and p53 have a potential to bind in the cytoplasm, but to a limited extent. Binding in HG condition is significantly enhanced, perhaps due to increased expression of both proteins in the cytoplasm, but still remains limited. We show for the first time that post-mitotic DAergic PC12 cells exert weak binding of GRP75 and p53, which contrasts with findings in nondifferentiated mitotic PC12 cells (Guo et al. 2009; Li et al. 2011). Altogether, our results demonstrate that HG-induced oxidative stress and apoptosis of DAergic PC12 cells can be improved by RESV, sustaining an important role for this naturally occurring polyphenol in diabetes treatment. RESV has been the object of several diabetes studies because of its ability to improve insulin sensitivity, protect pancreatic β cells, and control glycaemia (Milne et al. 2007; Szkudelski and Szkudelska 2011; Lee et al. 2012). Indeed, RESV protects against retinopathy in rats with diabetes (Soufi et al. 2012) and prevents nephropathy in db/db mice by inhibiting lipotoxicity-related apoptosis and oxidative stress in the kidney (Kim et al. 2013). Additional beneficial effects of the stilbene RESV may contribute to alleviate obesity-induced metabolic complications (Rosenow et al. 2012) often related with diabetes. A recent clinical study has found oral administration of RESV to be effective in improving glycaemia in type 2 diabetes mellitus (Bhatt et al. 2012).

Even though RESV is principally metabolized into its glucoronide and sulfate conjugates, recent data show that these metabolites may possess beneficial properties (Delmas et al. 2011). Increased bioavailability due to a synergistic effect with other polyphenols or compounds, such as curcumin or the glycemic control drug metformin, must also be taken into account (Bruckbauer and Zemel 2013; Du et al. 2013). Besides, recent pharmacological advances have improved bioavailability of RESV (for details see Amiot et al. 2013; Neves et al. 2013). Finally, the potential beneficial properties of RESV on human health are broadly displayed in the literature and justify the need to further unravel the powerful cellular role of this dietary polyphenol.
